# Tunable Dirac points and high spin polarization in ferromagnetic-strain graphene superlattices

**DOI:** 10.1038/s41598-017-14948-y

**Published:** 2017-11-07

**Authors:** Qing-Ping Wu, Zheng-Fang Liu, Ai-Xi Chen, Xian-Bo Xiao, Guo-Xing Miao

**Affiliations:** 1 Department of Applied Physics, East China Jiaotong University, Nanchang, 330013 China; 2 Institute for Quantum Computing, University of Waterloo, Waterloo, ON N2L 3G1 Canada; 3 Department of Physics, Zhejiang Sci-Tech University, Hangzhou, 310018 China; 4 School of Computer Science, Jiangxi University of Traditional Chinese Medicine, Nanchang, 330004 China

## Abstract

Spin-dependent energy bands and transport properties of ferromagnetic-strain graphene superlattices are studied. The high spin polarization appears at the Dirac points due to the presence of spin-dependent Dirac points in the energy band structure. A gap can be induced in the vicinity of Dirac points by strain and the width of the gap is enlarged with increasing strain strength, which is beneficial for enhancing spin polarization. Moreover, a full spin polarization can be achieved at large strain strength. The position and number of the Dirac points corresponding to high spin polarization can be effectively manipulated with barrier width, well width and effective exchange field, which reveals a remarkable tunability on the wavevector filtering behavior.

## Introduction

Graphene has attracted enormous attention from experimentalists and theorists since its discovery. In particular, the high carrier mobility and small spin-orbit coupling in graphene make it very promising for applications in nanoelectronics and spintronics. Recently, it is theoretically predicted that^1,2^ depositing a ferromagnetic insulator (FI) such as EuO on graphene can induce an exchange proximity interaction^1,3^, and the exchange proximity interaction can be treated as an effective exchange field (EEF). The deposition of EuO on graphene has been experimentally realized and its proximity induced ferromagnetization has been confirmed^4^. Many theoretical works on spin transport through ferromagnetic graphene suggest that the spin current can be controlled by gate voltages^1,2,5^, magnetic barriers^6,7^, and local strain^8–10^. Particularly, Dell’Anna found that an inhomogeneous perpendicular magnetic field together with a strong in-plane spin splitting can produce a wavevector-dependent spin-filtering effect^6^. Zhai showed that ferromagnetic graphene junctions with a modulated substrate strain can achieve a strain-tunable spin current^8^. Recently, Wu has examined that the ferromagnetic graphene system combined with strain or Rashba spin-orbit coupling, or both, can induce a spin band gap and achieve complete spin polarization^10^.

At the same time, graphene superlattices with electrostatic potential or magnetic barrier have also received broad theoretical and experimental investigations^11–16^. In electrostatic potential graphene superlattices, a new Dirac point appears in the band structures^13,14^ and it’s exactly located at zero-averaged wave number (zero-$\bar{k}$)^15^. The zero-$\bar{k}$ gap associated with this new Dirac point is insensitive to both lattice constant and structural disorder, resulting in more controllable electronic transport in graphene superlattices. Extra Dirac points in the band structure at zero-$\bar{k}$ have been experimentally observed^14,17,18^. As a comparison, in magnetic graphene superlattices, new finite-energy Dirac points are generated in the band structure and the Fermi velocity at zero energy Dirac points is isotropically renormalized^19–21^. Recently, resonant tunneling in ferromagnetic graphene superlattices has been studied and its splitting in the transmission gap can be used to generate an efficient wavevector filter^22^. However, ferromagnetic graphene superlattices alone cannot suppress the spin-dependant Klein tunneling^23^, which results in finite spin polarization.

In addition, the pseudo magnetic field induced by the strain is an efficient method to suppress Klein tunneling^10,23^, and a local strain can be achieved by patterning grooves, creases, steps, or wells in the substrate where graphene rests^24–26^, so that different regions of the substrate interact differently with the graphene sheet, generating different strain profiles^27^. Evidence for strain-induced spatial modulations in the local conductance of graphene on SiO_2_ substrates has already been reported in experiment^28^. Building from these literature works, we now consider a ferromagnetic-strain graphene superlattice, where the spin-dependant Klein tunneling is violated. We first discuss the zero-$\bar{k}$ and finite-energy Dirac points’ locations in the spectrum of a ferromagnetic graphene superlattices in detail. When strain is also considered, we observe that a band gap is induced in the vicinity of finite-energy Dirac points, and the band gaps for spin-up and spin-down electrons are present in different energy regions. The spin-dependent band structure is clearly reflected in the transport properties, which provides a guide for enhancing the spin polarization. The position and number of Dirac points, and the corresponding high spin polarization, can be effectively manipulated by adjusting the barrier width, well width and EEF strength, which demonstrates remarkable tunability on the wavevector filtering behavior.

The paper is organized as follows. In Sec. II, we present the theoretical formalisms and the dispersion relations. The numerical results on band structures and transmission for different spins are shown in Secs. III. Finally, we draw conclusions in Sec. IV.

### Computational Models and Methods

**Figure 1:**
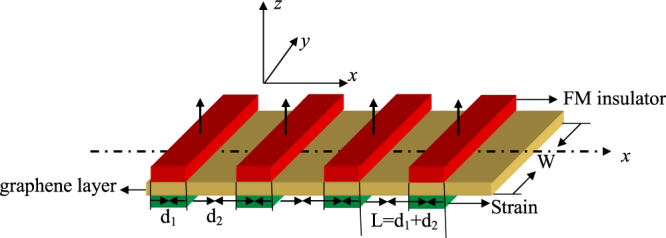
Schematic illustration of the Ferromagnetic-strained graphene superlattices produced by a series of FM stripes and substrate strains. *W* is the width of the graphene sample in the *y* direction. The length of one unit is *L* = *d*_1_ + *d*_2_, *d*_1_ is the width of the Ferromagnetic-strained graphene, *d*_2_ is the width of the normal graphene.

Let us consider a one-dimensional ferromagnetic-strained superlattice in graphene formed by a series of EEF barriers and strained barriers. In our case, we consider a series of FI strips with *z*-axis magnetizations deposited periodically on the top of graphene to induce the EEF barriers^1,3^. It has been demonstrated that the EEF between electrons in graphene and localized electrons in an adjacent FI layer is about 5 meV^1^ and can be further enhanced by applying an external electric field perpendicular to the graphene sheet^3^. In this paper, the local strains are assumed inside these FI stripe regions, which can be induced by a tension along the *y* direction applied on the substrate rather than the graphene. It is known that graphene can sustain elastic up to 25%^29,30^. The elastic deformation can be treated as a perturbation to the hopping amplitudes and acts as a pseudogauge potential *A*_*s*_(*r*)^31,32^. Here the pseudogauge potential is induced by the uniaxial strain. As a corollary, the pseudogauge potential is a finite and constant, which is defined as *As*(*r*) = *As*(*x*) = *tβε*(1 + *σ*)^33^, and *σ* = 0.165 is the Poisson’s ratio of graphite, *t* is the nearest-neighbour hopping parameter, and *ε* is the tensile strain. The constant *β* = ∂*lnt*/∂*lnδ*, where *δ* is the distance between nearest carbon atoms. Several units of such structures are depicted in Fig. 1, and the length of each unit is *L* = *d*_1_ + *d*_2_. The low-energy effective Hamiltonian for ferromagnetic-strain graphene can be written as

1 $$H(r)=-i\hslash {v}_{F}({\tau }_{z}{\sigma }_{x}{\partial }_{x}+{\sigma }_{y}{\partial }_{y})+{\tau }_{z}{A}_{s}(x){\sigma }_{y}+M{s}_{z},$$

where *v*_*F*_ ≈ 10^6^ m/s is the Fermi velocity, *τ*_*z*_ = ±1 for *K* and *K* ^'^ valleys, *σ*_*i*_ and *s*_*i*_ (*i* = *x*, *y*, *z*) are the Pauli matrices acting on the sublattice (*A*, *B*) and physical spin (↑, ↓) spaces, respectively. Due to the translational invariance in the *y* direction, the wave function in the *j* th ferromagnetic-strained barrier can be presented as $\tilde{{\rm{\Psi }}}={\rm{\Psi }}(x){e}^{i{k}_{y}x}$ with

2 $${\rm{\Psi }}(x)=[{a}_{j}{e}^{i{q}_{j}x}(\frac{\begin{array}{c}1\\ {q}_{j}+i{k}_{yj}\end{array}}{{k}_{j}})+{b}_{j}{e}^{-i{q}_{j}x}(\frac{\begin{array}{c}1\\ -{q}_{j}+i{k}_{yj}\end{array}}{{k}_{j}})],$$

where ${k}_{j}=\tfrac{(E-s{M}_{j})}{(\hbar {v}_{F})}$ (*s* = ±1 for spin-up and spin-down electrons) and *k*_*yj*_ = *k*_*y*_ + *τ*_*z*_(*A*_*sj*_)/(ħ*v*_*F*_). *k*_*y*_ = (*Esinθ*_0_)/(ħ*v*_*F*_), *θ*_0_ is the incident angle. *q*_*j*_ = sign (*k*_*j*_) (*k*_*j*_^2^ − *k*_*yj*_^2^)^1/2^ for *k*_*j*_^2^ > *k*_*yj*_^2^, otherwise *q*_*j*_ = sign(*k*_*j*_) *i*(*k*_*yj*_^2^ − *k*_*j*_^2^)^1/2^, and *a*_*j*_(*b*_*j*_) is the amplitude of the forward (backward) propagating wave. For the well region, the above equations are still valid and only require that *M*_*j*_ = 0 and *A*_*sj*_ = 0. Inside the same barrier or well region, the wave functions at any two positions *x* and *x* + Δ*x* can be related via the transfer matrix^16^

3 $${{\rm{\Gamma }}}_{j}({\rm{\Delta }}x,E,{k}_{y})=(\begin{array}{cc}\frac{\cos ({q}_{j}{\rm{\Delta }}x-{\theta }_{j})}{\cos \,{\theta }_{j}} & i\frac{\sin ({q}_{j}{\rm{\Delta }}x)}{\cos \,{\theta }_{j}}\\ i\frac{\sin ({q}_{j}{\rm{\Delta }}x)}{\cos \,{\theta }_{j}} & \frac{\cos ({q}_{j}{\rm{\Delta }}x+{\theta }_{j})}{\cos \,{\theta }_{j}}\end{array}),$$

with *θ*_j_ = arcsin(k_yj_/k_j_). Furthermore, the overall *T*-matrix for the *N* regions is simply a product of matrices:

$$X=[\begin{array}{cc}{X}_{11} & {X}_{12}\\ {X}_{21} & {X}_{22}\end{array}]=\prod _{j=1}^{N}{{\rm{\Gamma }}}_{j}({w}_{j},E,{k}_{y})\mathrm{.}$$

Here the *w*_*j*_ is the width of the *j*th potential region. And we can connect the input and output wave functions by the relation: Ψ(*x*_*N*_) = *X*Ψ(*x*_0_), where the Ψ(*x*_*N*_) and Ψ(*x*_0_) can be written as:^15^

$${\rm{\Psi }}({x}_{0})=(\begin{array}{c}1+{r}_{s,{\tau }_{z}}\\ ({e}^{i{\theta }_{0}}-{r}_{s,{\tau }_{z}}{e}^{-i{\theta }_{0}})\end{array})\,{{\rm{\Psi }}}_{i}(E,{k}_{y}),$$

and

$${\rm{\Psi }}({x}_{N})=(\begin{array}{c}{t}_{s,{\tau }_{z}}\\ {t}_{s,{\tau }_{z}}{e}^{i{\theta }_{N}}\end{array})\,{{\rm{\Psi }}}_{i}(E,{k}_{y}\mathrm{).}$$

Here, *θ*_*N*_ is the exit angle at the exit end, Ψ_*i*_(*E*, *k*_*y*_) is the incident wave packet of the electron, ${r}_{s,{\tau }_{z}}$ is the spin/valley resolved reflection coefficient and *t*_s,τz_ is the spin/valley resolved transmission coefficient, respectively. Solving the above two equations, we find the *r*_s,τz_ and *t*_s,τz_ can be given

4 $$\begin{array}{rcl}{r}_{s,{\tau }_{z}} & = & \frac{({X}_{22}{e}^{i{\theta }_{0}}-{X}_{11}{e}^{i{\theta }_{N}})-{X}_{12}{e}^{i({\theta }_{0}+{\theta }_{N})}+{X}_{21}}{({X}_{22}{e}^{-i{\theta }_{0}}+{X}_{11}{e}^{i{\theta }_{N}})-{X}_{12}{e}^{i({\theta }_{N}-{\theta }_{0})}-{X}_{21}},\\ {t}_{s,{\tau }_{z}} & = & \frac{2\,\cos \,{\theta }_{0}}{({X}_{22}{e}^{-i{\theta }_{0}}+{X}_{11}{e}^{i{\theta }_{N}})-{X}_{12}{e}^{i({\theta }_{N}-{\theta }_{0})}-{X}_{21}}\mathrm{.}\end{array}$$

Once the transmission coefficient is obtained, the spin/valley resolved conductance *G*_*s*,*τz*_ of the system at zero temperature is written as *G*_s,τz_ = *G*_0_∫_0_^(*π*)/(2)^ *T*_s,τz_ *cosθ*_0_ *dθ*_0_, where *T*_s,τz_ = |*t*_s,τz_^2^|, *G*_0_ = 2*e* ^2^ *mv*_*F*_ *L*_*y*_/*ħ* ^*2*^ and *L*_*y*_ is the width of the graphene stripe in the *y* direction. Meanwhile, the spin polarizations are defined as

5 $${P}_{{\tau }_{z}}=\frac{{G}_{\uparrow ,{\tau }_{z}}-{G}_{\downarrow ,{\tau }_{z}}}{{G}_{\uparrow ,{\tau }_{z}}+{G}_{\downarrow ,{\tau }_{z}}}\mathrm{.}$$

## Results and Discussion

In order to understand the transport properties, it is instructive to first investigate the electronic band structure for the ferromagnetic-strain graphene supperlatice. According to the Bloch’s theorem^15^, the electronic dispersion for any transversal wave number follows the relation:

6 $$\begin{array}{rcl}\cos \,[{K}_{s,{\tau }_{z}}L] & = & \frac{1}{2}Tr({{\rm{\Gamma }}}_{1}\,{{\rm{\Gamma }}}_{2})\\  & = & \cos ({q}_{1}{d}_{1})\,\cos ({q}_{2}{d}_{2})\\  &  & +\frac{{k}_{y}({k}_{y}+{\tau }_{z}\frac{{A}_{s}}{\hslash {v}_{F}})-\frac{E(E-sM)}{{(\hslash {v}_{F})}^{2}}}{{q}_{1}{q}_{2}}\,\sin ({q}_{1}{d}_{1})\,\sin ({q}_{2}{d}_{2})\end{array}.$$

Here *K*_*s*,*τz*_ is Bloch wave vector. Γ_1_ and Γ_2_ are the transfer matrixes for one barrier and one well, respectively. *q*_1_ = sign(*E* − *sM*)$\sqrt{{(\tfrac{E-{}_{s}M}{\hslash {v}_{F}})}^{2}\,-\,{({k}_{y}+{\tau }_{z}\tfrac{{A}_{s}}{\hslash {v}_{F}})}^{2}}$ for ${(\frac{E-sM}{\hslash vF})}^{2} > {({k}^{y}+{\tau }_{z}\tfrac{As}{\hslash {v}_{F}})}^{2}$, otherwise ${q}_{1}={\rm{sign}}(E-sM)i$ $\sqrt{{({k}_{y}+{\tau }_{z}\tfrac{{{\rm{A}}}_{s}}{\hslash {v}_{F}})}^{2}-{(\tfrac{E-sM}{\hslash {v}_{F}})}^{2}}$. ${q}_{2}={\rm{sign}}(E)\sqrt{\tfrac{{E}^{2}}{{(\hslash {v}_{F})}^{2}}+{k}_{y}^{2}}\,{\rm{for}}\,\tfrac{{E}^{2}}{{(\hslash {v}_{F})}^{2}} > {{k}_{y}}^{2}$, otherwise ${q}_{2}={\rm{sign}}(E)i\sqrt{{k}_{y}^{2}-\tfrac{{E}^{2}}{{(\hslash {v}_{F})}^{2}}}$. Using |cos(*K*_s,τz_ *L*)| ≤ 1, we can find the real solution of ${K}_{s,{\tau }_{z}}$ for passing band_*s*_. Otherwise, the non-existence of real ${K}_{s,{\tau }_{z}}$ indicates a band gap^34^.

**Figure 2:**
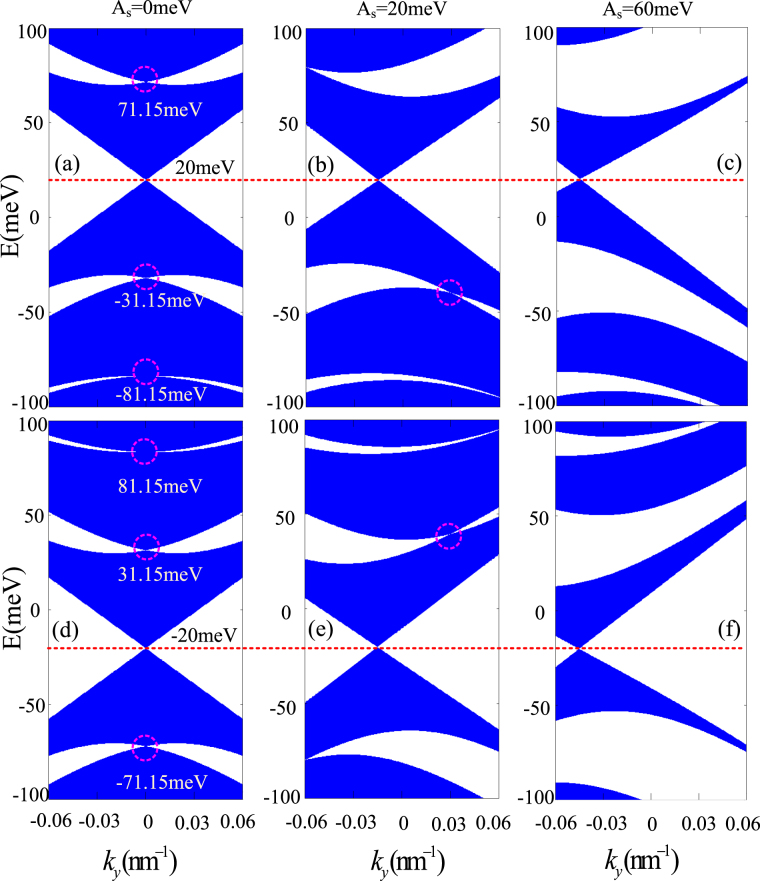
Electronic band structures for up spin (**a**–**c**) and down spin (**d**–**f**) with different strain strength: (**a**) and (**d**) *A*_*s*_ = 0 meV; (**b**) and (**e**) *A*_*s*_ = 20 meV; (**c**) and (**f**) *A*_*s*_ = 60 meV; The dotted lines denote the centre position of the zero-averaged wave number Dirac point. The round dashed lines denote the centre position of the finite-energy Dirac points. The other parameters are *M* = 40 meV, *d*_1_ = *d*_2_ = 20 nm.

Now let us use the above equations to calculate the electronic band structures under different strain strength. The transmissions of electrons in *K* and *K* ^'^ valleys show mirror symmetry^10,23^, so we focus only on the spin transport for the valley *K*. When *A*_*s*_ = 0 (Fig. 2a,d), we find that the zero-$\bar{k}$ Dirac point is given at $E=\frac{sM}{2}$, *k*_*y*_ = 0. When strain is considered (Fig. 2b,c,e,f), we find that the zero-$\bar{k}$ Dirac point is shifted to ${k}_{y}=-\frac{{A}_{s}}{2\hslash {v}_{F}}$ along $E=\frac{sM}{2}$. Here $\hslash {v}_{F}=(\frac{4.14\times {10}^{-12}}{2\pi }\,{\rm{meV}}\cdot {\rm{s}})\,({10}^{15}\,{\rm{nm}}/{\rm{s}})=\frac{2070}{\pi }\,{\rm{meV}}\cdot {\rm{nm}}$. In other words, the Dirac point shifts to ${k}_{y}=-\frac{{A}_{s}}{2\hslash {v}_{F}}$ in *k* space, but the energy is invariable. Such a result can be solved by the dispersion relation of Eq. (6).

Applying the implicit function theorem, the gradient of the dispersion relation will be zero only if sin(*q*_1_ *d*_1_) = sin(*q*_2_ *d*_2_) = 0 and cos(*q*_1_ *d*_1_) = cos(*q*_2_ *d*_2_) = 1. When *A*_*s*_ = 0, the following equations are satisfied

7 $$\begin{array}{rcl}{q}_{1}{d}_{1} & = & sign(E\,-\,sM){[{(\frac{E-sM}{\hslash {v}_{F}})}^{2}-{k}_{y}^{2}]}^{\frac{1}{2}}{d}_{1}=m\pi ,\\ {q}_{2}{d}_{2} & = & sign(E){[{(\frac{E}{\hslash {v}_{F}})}^{2}-{{k}_{y}}^{2}]}^{\frac{1}{2}}{d}_{2}=n\pi \mathrm{.}\end{array}$$

Here *m*, *n* are integers. The equation (6) shows that *cos*(*K*_s,τz_ *L*) = *cos*(*q*_1_ *d*_1_ ± *q*_2_ *d*_2_) for *k*_*y*_ = 0 and *A*_*s*_ = 0, which indicates that *K*_s,τz_ always has real solutions for any *E* and *M*; that is, the location of the crossing point of the bands exactly appears at *k*_*y*_ = 0. So under the condition of *k*_*y*_ = 0 and *A*_*s*_ = 0, one can get $E=\tfrac{sM{d}_{1}}{{d}_{1}+{d}_{2}}+\hslash {v}_{F}{\rm{\pi }}\tfrac{m+n}{{d}_{1}+{d}_{2}}$. If the condition *q*_1_ *d*_1_ =− *q*_2_ *d*_2_ = *mπ* is satisfied, the solution is $E=\tfrac{sM{d}_{1}}{{d}_{1}+{d}_{2}}$, which is the so-called zero-$\bar{k}$ Dirac point^15^. Further when *d*_1_ = *d*_2_, the zero-$\bar{k}$ Dirac point is located at $E=\tfrac{sM}{2}$, *k*_*y*_ = 0. This way, we can also locate the other crossing points corresponding to the finite-energy Dirac points^20^, at $E=\tfrac{sM{d}_{1}}{{d}_{1}+{d}_{2}}$ + $\hslash {v}_{F}\pi \frac{l}{{d}_{1}+{d}_{2}}$(*l* = ±1, ±2 $\cdots$) and *k*_*y*_ = 0. From Fig. 2a,d, one can observe that the finite-energy Dirac points for the spin-up (spin-down) band is exactly located in *E* = 71.15 meV, −31.15 meV, −81.15 meV (81.15 meV, 31.15 meV, −71.15 meV). Moreover, Fig. 2a,d also suggest that the spin-up and spin-down Dirac points don’t always coincide, which plays a key role in spin-dependent transport, but it is noted that the bands always cross at *k*_*y*_ = 0.

In addition, if *A*_*s*_ ≠ 0, one can find

8 $$\begin{array}{rcl}{q}_{1}{d}_{1} & = & {\rm{sign}}(E-sM){[{(\frac{E-sM}{\hslash {v}_{F}})}^{2}-{({k}_{y}+\frac{{A}_{s}}{\hslash {v}_{F}})}^{2}]}^{\frac{1}{2}}{d}_{1}=m\pi ,\\ {q}_{2}{d}_{2} & = & {\rm{sign}}(E){[{(\frac{E}{\hslash {v}_{F}})}^{2}-{{k}_{y}}^{2}]}^{\frac{1}{2}}{d}_{2}=n\pi \end{array}.$$

When *q*_1_ *d*_1_ =− *q*_2_ *d*_2_ = *mπ* is also satisfied, we can get

9 $$\sqrt{{(\frac{E{d}_{1}-sM{d}_{1}}{\hslash {v}_{F}})}^{2}-{({k}_{y}{d}_{1}+\frac{{A}_{s}}{\hslash {v}_{F}}{d}_{1})}^{2}}-\sqrt{{(\frac{E{d}_{2}}{\hslash {v}_{F}})}^{2}-{({k}_{y}{d}_{2})}^{2}}=0.$$

The equation (9) is tenable under the conditions *Ed*_1_ − *sMd*_1_ =−*Ed*_2_ and *k*_*y*_ *d*_1_ + $\frac{{A}_{s}}{\hslash {v}_{F}}$ *d*_1_ = −*k*_*y*_ *d*_2_, so *E* = $\frac{sM{d}_{1}}{{d}_{1}+{d}_{2}}$, ${k}_{y}=-\tfrac{As{d}_{1}}{\hslash {v}_{F}({d}_{1}+{d}_{2})}$ is one solution of Eq. (9), which corresponds to the zero-$\bar{k}$ Dirac point. If *d*_1_ = *d*_2_, zero-$\bar{k}$ Dirac point is located at $E=\tfrac{sM}{2}$, ${k}_{y}=-\tfrac{As}{2\hslash {v}_{F}}$. However, it is more difficult to find analytic solutions of the finite-energy Dirac points like the analytic results obtained by solving Eq. (7). But numerical calculations show that the finite-energy Dirac points are strongly affected by the strain strength. Due to the effects of strain, the finite-energy Dirac points are not only shifted in energy but also decreased in number (Fig. 2b,e), even disappear completely for large strain strengths (Fig. 2c,f). Then, there emerges an energy gap in the vicinity of the vanished finite-energy Dirac points with further increasing the strain strength. And the energy gaps for the spin-up and spin-down bands don’t fully overlap. These characters mean that the increasing of *A*_*s*_ may be used to enhance the spin polarization in ferromagnetic-strain graphene superlattices.

**Figure 3:**
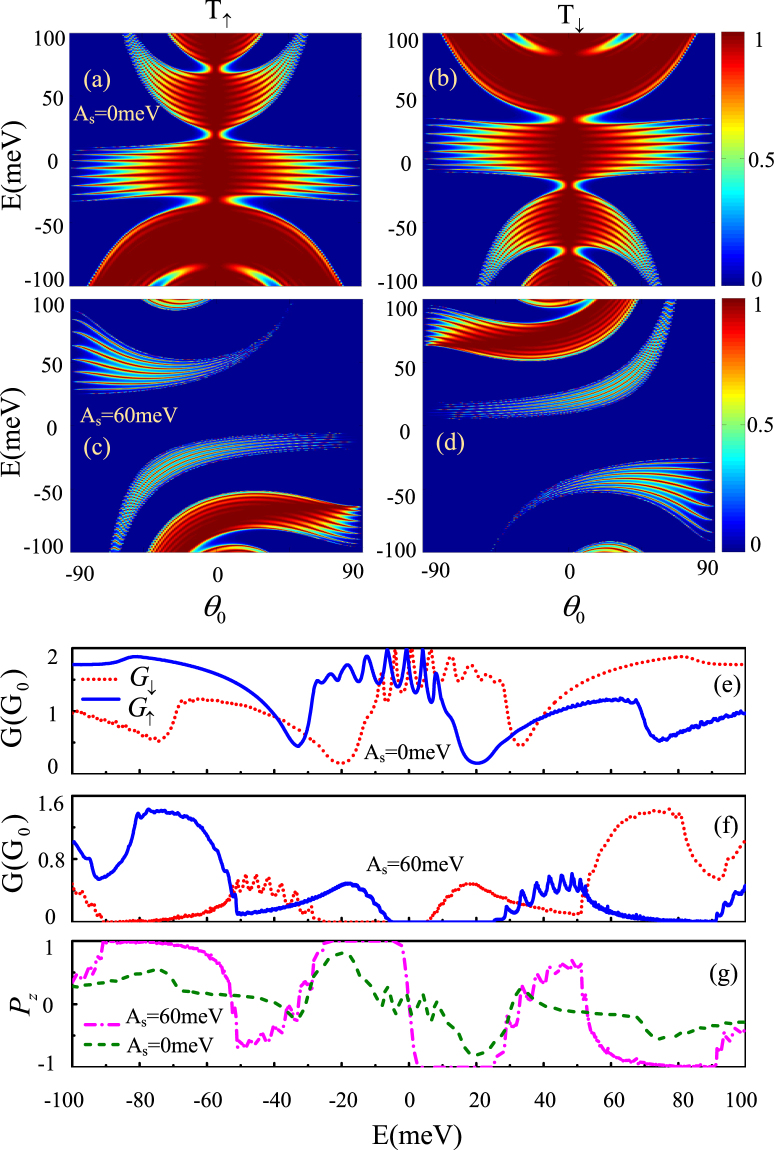
(**a**–**d**) Spin-dependent transmission *T*_*s*_ with different strain strength (**a**) and (**b**) *A*_*s*_ = 0 meV, (**c**) and (**d**) *A*_*s*_ = 60 meV versus Fermi energy and incident angle; (**e** and **f**) spin-dependent conductance *G*_*s*_ and (**g**) spin polarization *P*_*z*_ versus Fermi energy. the periodic number *N* = 10, and *M* = 40 meV, *d*_1_ = *d*_2_ = 20 nm.

The above discussions on the band structures should be helpful for understanding the spin-dependent transport. Figure 3 displays the spin-dependent transmission *T*_*s*_, spin-dependent conductance *G*_*s*_ and spin polarization along *z* direction *P*_*z*_ of the ferromagnetic-strain graphene superlattices under different strain. Here we only consider *A*_*s*_ = 0 and *A*_*s*_ = 60 meV, and take the superlattice period number *n* = 10. In the absence of strain (Fig. 3a,b), the transmission shows a spin-dependent Klein tunneling and embodies the mirror symmetry about *θ* = 0. But the transmission for up-spins is different from that for down-spins, especially at the locations of the Dirac points where the transport channels for up-(down-) spins are finite, while the transport channels for down-(up-) spins are large. These characters ensure that the two spin conductance channels are obviously different at these Dirac points (as seen in Fig. 3e), and finite spin polarization appears (as seen in Fig. 3(g)). When strain is considered (Fig. 3c,d), we find that the mirror symmetry with *θ* = 0 is destroyed because of the shifted Dirac points by the strain, and the spin-dependent Klein tunneling is suppressed due to the spin-dependent band gap induced by the strain. It is noted that the spin-dependent transmission gaps also induce zero-$\bar{k}$ Dirac points nearby because the spin-dependent waves inside the potential barrier are evanescent waves when the relation ${(E-sM)}^{2} < {({k}_{y}+{\tau }_{z}{A}_{s})}^{2}$ is satisfied. Then we find that the spin-up and spin-down conductances are totally different around those disappeared Dirac points. Especially, in the vicinity of *E* = 20 meV, 71.15 meV (*E* =−20 meV, −71.15 meV), the spin-depended conductance *G*_↓_ (*G*_↑_) shows a broad peak, while *G*_↑_ (*G*_↓_) approaches zero (Fig. 3f), so fully spin polarized plateaus with large spin-polarized currents are achieved around these Fermi energies (as seen in Fig. 3g). In addition, spin polarization oscillations are obtained as seen in Fig. 3g, which can be used as a spin switch by modulating the Fermi energy.

**Figure 4:**
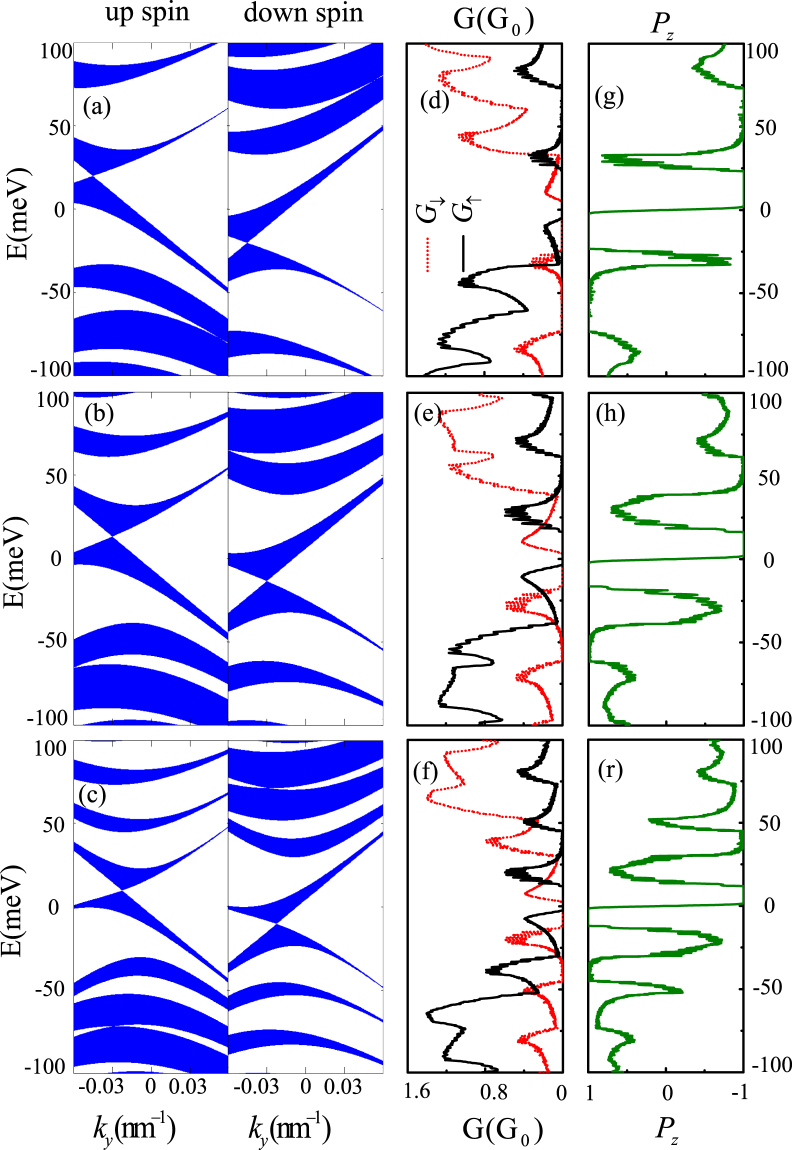
(**a**–**c**) Electronic band structures for up spin and down spin for *M* = 40 meV with different barrier and the well width: (**a**) *d*_1_ = *d*_2_ = 30 nm; (**b**) *d*_1_ = 20 nm, *d*_2_ = 40 nm; (**c**) *d*_1_ = 20 nm, *d*_2_ = 60 nm; (**d**–**f**) spin-dependent conductance *G*_*s*_ and spin polarization *P*_*z*_ versus Fermi energy with the periodic number *N* = 10 corresponds to the cases in (**a**–**c**).

The above discussions show that high spin polarizations always appear in the vicinity of the vanished Dirac points. And the positions of Dirac points in (*E*, *k*_*y*_) space can be controlled by the barrier and well widths. Figure 4a–c show the band structures with different barrier and well widths. The locations of the zero-$\bar{k}$ Dirac points move towards *E* = 0 and *k*_*y*_ = 0 with gradually reduced *d*_1_/(*d*_1_ + *d*_2_) ratio at fixed heights of potentials. The locations of the band gaps around the vanished finite-energy Dirac points move toward *E* = 0 too. In addition, the number of band gaps increases with the increase of the lattice constant *d*_1_ + *d*_2_. The reason of that is the zero-$\bar{k}$ Dirac points is exactly located $E=\tfrac{sM{d}_{1}}{{d}_{1}+{d}_{2}}$, ${k}_{y}=-\tfrac{As{d}_{1}}{{d}_{1}+{d}_{2}}$, which is determined by the *d*_1_/(*d*_1_ + *d*_2_) ratio. The finite-energy Dirac points are located at $E=\tfrac{sM{d}_{1}}{{d}_{1}+{d}_{2}}+\hslash {v}_{F}{\rm{\pi }}\tfrac{l}{{d}_{1}+{d}_{2}}$, which depends not only on the *d*_1_/(*d*_1_ + *d*_2_) ratio but also the lattice constant *d*_1_ + *d*_2_. So we can modulate the location and number of high spin polarization regions by adjusting the the *d*_1_/(*d*_1_ + *d*_2_) ratio and the lattice constant.

Next we consider the spin-dependent conductance *G*_*s*_ (Fig. 4d–f) and spin polarization *P*_*z*_ (Fig. 4h–r) of an electron passing through the ferromagnetic-strain graphene superlattices with different width. Comparison between Fig. 4a–f indicates that the distribution of transmission spectra is completely consistent with the band structures, that is, strong transmission regions correspond to the transmission bands and forbidden transmission regions correspond to the band gaps. Then, the location of high spin polarization approaches *E* = 0 with the decrease of *d*_1_/(*d*_1_ + *d*_2_) ratio, and the number of high spin polarization regions increases with increasing lattice constants (Fig. 4h–r). Therefore the increase of lattice constants makes the spin polarization oscillations more obvious.

**Figure 5:**
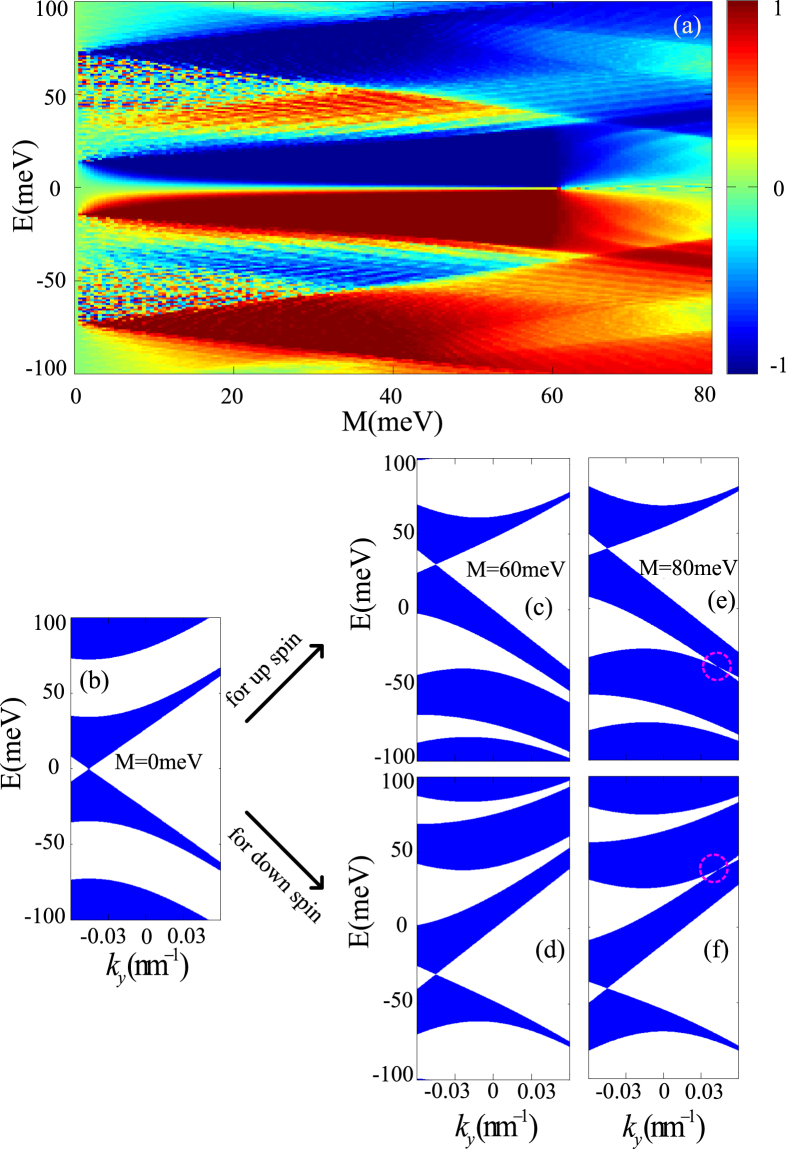
(**a**) spin polarization *P*_*z*_ versus Fermi energy and exchange field strength with the periodic number *N* = 10, (**b**–**f**) Electronic band structures for up spin and down spin with different exchange field strength: (**a**) *M* = 0 meV; (**c**) and (**d**) *M* = 60 meV; (**e** and **f**) *M* = 80 meV; The other parameters are *d*_1_ = *d*_2_ = 20 nm, *A*_*s*_ = 60 meV.

In addition, the height of potentials can also affect the locations of the Dirac points. Figure 5a shows the spin polarization with respect to *M* and *E* for *d*_1_ = *d*_2_ = 20 nm. In the absence of EEF, the spin polarization is zero (Fig. 5a) due to the spin degeneracy (as seen in Fig. 5b). And the spin polarization initially increases and then decreases with increasing the EEF strength for *M* ≥ *A*_*s*_ (Fig. 5a). The reason is that when the EEF strength *M* ≥ *A*_*s*_, the gaps around the Dirac points are finite (as seen in Fig. 5c,d) and the crossing points even reappear for larger *M* (as seen in Fig. 5e,f), which leads to both up-spins and down-spins having transport channels around the Dirac points therefore the spin polarization is reduced. So too large *M* does not guarantee effective spin filtering in such ferromagnetic-strain graphene superlattices. We also find that the high spin polarization regions are shifted away from zero energy owing to the shift of Dirac points away from *E* = 0 with the increasing of EEF.

## Summary

In summary, we studied the spin-dependent band structures and transport properties of graphene under a periodic effective exchange field and strain, where the spin-dependant Klein tunneling is disrupted. We discussed the zero wave number Dirac points’ and finite-energy Dirac points’ locations on the spectra of ferromagnetic graphene superlattices in detail. The spin-up and spin-down Dirac points are present on the energy spectra alternately, which results in finite spin polarization. When strain is considered, band gaps are induced around the finite-energy Dirac points, and high spin polarization is achieved in the vicinity of these Dirac points. The position, and number of the Dirac points can be effectively manipulated by adjusting the barrier width, well width and EEF strength, which leads to tunable spin polarization. We hope these results are helpful for understanding the electronic properties for spin transports and can offer guidance to potential applications of the spin filtering devices.
